# Association Between the *ANGPT2* rs2442598 Polymorphism and Diabetic Nephropathy in Slovenian Patients with Type 2 Diabetes Mellitus

**DOI:** 10.3390/genes17040373

**Published:** 2026-03-25

**Authors:** Petra Nussdorfer, Jernej Letonja, Matej Završnik, Boštjan Matos, Danijel Petrovič, Ines Cilenšek

**Affiliations:** 1 Laboratory for Histology and Genetics of Atherosclerosis and Microvascular Diseases, Faculty of Medicine, University of Ljubljana, Korytkova 2, 1000 Ljubljana, Slovenia; petra.nussdorfer@mf.uni-lj.si (P.N.); jernej.letonja@mf.uni-lj.si (J.L.); danijel.petrovic@mf.uni-lj.si (D.P.); 2 Institute of Histology and Embryology, Faculty of Medicine, University of Ljubljana, Korytkova 2, 1000 Ljubljana, Slovenia; 3 Department of Endocrinology and Diabetology, Internal Medicine Clinic, University Clinical Center, 2000 Maribor, Slovenia; matej.zavrsnik@ukc-mb.si; 4 Department of Neurosurgery, University Clinical Center, 1000 Ljubljana, Slovenia; bostjan.matos@kclj.si

**Keywords:** diabetic nephropathy, type 2 diabetes mellitus, *ANGPT2*, *VEGFA*, polymorphism

## Abstract

**Background:** The aim of our study was to evaluate the association of angiopoietin 2 (*ANGPT2*) rs2442598 and vascular endothelial growth factor A (*VEGFA*) rs2010963 with diabetic nephropathy (DN) in Slovenian subjects with type 2 diabetes mellitus (T2DM). Angiopoietin–endothelial tyrosine kinase receptor (Ang-Tie2) and VEGF-A signaling regulate glomerular endothelial stability and permeability and may contribute to DN susceptibility. **Methods:** We conducted a case–control study including 897 unrelated Slovenian subjects with T2DM (344 DN cases; 553 long-standing T2DM controls without DN). *ANGPT2* rs2442598 and *VEGFA* rs2010963 were genotyped using TaqMan assays. Genetic associations were analysed using co-dominant, additive, dominant, and recessive genetic models with logistic regression adjusted for waist circumference, systolic blood pressure, fasting glucose, and triglycerides. **Results:** *ANGPT2* rs2442598 was significantly associated with DN, with increased risk in carriers of the C allele, including a significant additive per allele effect (OR 1.39, 95% CI 1.10–1.74) and a dominant model effect (OR 1.47, 95% CI 1.11–1.96). In contrast, *VEGFA* rs2010963 showed no evidence of association across genetic models. **Conclusions:** In Slovenian patients with T2DM, *ANGPT2* rs2442598 is associated with DN, whereas *VEGFA* rs2010963 is not. This association suggests that *ANGPT2* genetic variation may influence DN risk and supports further functional work to define the biological effects of rs2442598.

## 1. Introduction

Diabetes mellitus (DM) is a chronic, multifactorial metabolic disease characterized by impaired insulin secretion and insulin action, leading to persistent hyperglycaemia and an increased risk of microvascular and macrovascular complications [1,2,3]. Recent global estimates indicate that 11.1% (approximately 1 in 9) of adults aged 20–79 years live with diabetes, and over 90% of cases are type 2 diabetes mellitus (T2DM); the rising prevalence is largely driven by urbanisation, population ageing, physical inactivity, and increasing overweight and obesity [1,2,3,4,5,6,7,8].

Diabetic kidney disease (DKD), commonly referred to as diabetic nephropathy (DN), is one of the most common microvascular complications of T2DM and a leading cause of end-stage kidney disease (ESKD) [3]. Approximately 25–30% of individuals with long-standing T2DM develop DN [1,2,9]. Evaluation of renal involvement relies on biochemical markers, including urinary albumin excretion and estimated glomerular filtration rate (eGFR), and, when indicated, structural assessment using imaging and kidney biopsy [10,11,12]. The earliest clinically detectable abnormality is persistent microalbuminuria (30–300 mg/day), reflecting early glomerular injury. Cross-sectional studies report microalbuminuria in approximately 28–44% of patients with T2DM and overt proteinuria in 7–8% [4,12,13]. Without appropriate treatment, a proportion of patients progress to overt DN and may ultimately develop ESKD [3,5,10,11,12,13,14].

Histopathological studies show that biochemical markers of DN are accompanied by progressive structural alterations in the glomeruli and tubulointerstitium [15,16]. Early lesions include glomerular hyperfiltration and hyperperfusion, followed by thickening of the glomerular basement membrane (GBM), mesangial expansion, and glomerular hypertrophy [16]. Continued metabolic and haemodynamic injury can lead to podocyte loss, tubulointerstitial inflammation and fibrosis, and a progressive reduction in functional nephron number, culminating in kidney failure in advanced disease [15,16,17]. DN is a complex multifactorial complication with a strong genetic component, and genetic susceptibility influences both its development and progression [8,10,14]. Therefore, genes involved in angiogenic signalling, such as *ANGPT2* and *VEGFA*, are plausible candidates for DN risk and progression [14,18,19].

**Figure 1:**
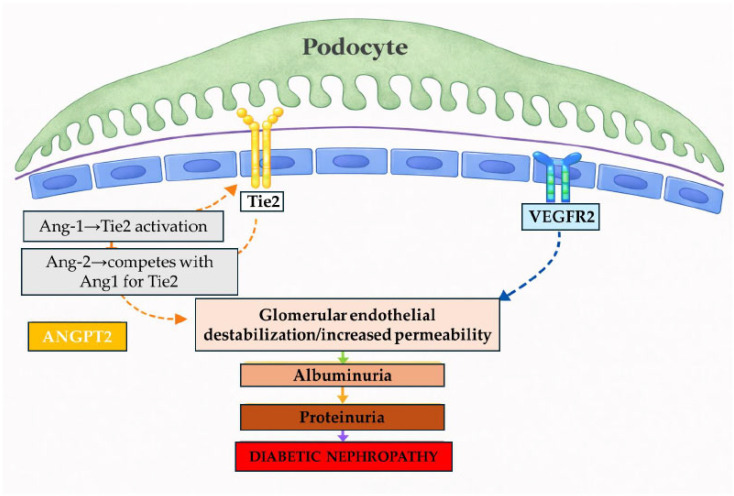
The proposed role of Ang-2-Tie2 and VEGF-A signaling in diabetic nephropathy. VEGF-A, released by podocytes, signals through VEGFR2 on glomerular endothelial cells and contributes to endothelial homeostasis (blue dashed arrow), whereas Ang-1/Tie2 signaling stabilizes the endothelium and Ang-2 antagonizes this pathway (orange dashed arrows). Dysregulation of these pathways may contribute to endothelial destabilisation, increased permeability, albuminuria, proteinuria, and the development of diabetic nephropathy, as indicated by the solid downward arrows. Legend: Ang-1: angiopoietin-1; Ang-2: angiopoietin-2; Tie2: endothelial tyrosine kinase receptor; VEGF-A: vascular endothelial growth factor A; VEGFR2: vascular endothelial growth factor receptor 2.

Angiopoietins (ANGPTs) are endothelial growth factors that regulate angiogenesis, vasculogenesis, and vascular remodelling by binding the endothelial tyrosine kinase receptor Tie2 (TEK) [18,19]. The two principal ligands, angiopoietin-1 (Ang-1) and angiopoietin-2 (Ang-2), exert largely opposing effects. Ang-1 activates Tie2 signalling and promotes endothelial stability by strengthening intercellular junctions and maintaining vessel integrity [20,21]. In contrast, Ang-2 competes with Ang-1 for Tie2 binding, thereby attenuating Ang-1-mediated Tie2 phosphorylation, and can also interact with integrins, promoting endothelial destabilisation and apoptosis under pathological conditions [20,22,23,24]. In individuals with T2DM, circulating Ang-2 levels are elevated compared with non-diabetic controls and correlate positively with albuminuria and the urinary albumin-to-creatinine ratio, consistent with renal microvascular injury [25,26,27], as illustrated in Figure 1.

The *ANGPT2* gene is located on chromosome 8p23.1, and rs2442598 is an intronic variant [27]. Only a few association studies have examined rs2442598 in diabetes-related microvascular outcomes, and evidence for DN in T2DM remains limited. The most consistent diabetes-specific association to date has been reported for diabetic retinopathy in type 1 diabetes [28]. In addition, rs2442598 has been linked to ESKD in a case–control study; however, ESKD included all causes, so it was not specific to DN. Beyond diabetes-related renal outcomes, *ANGPT2* genetic variation has been associated with cardiovascular and inflammatory conditions, including coronary artery disease [29] and systemic sclerosis [30]. Associations have also been reported in trauma-associated acute lung injury with altered circulating Ang-2 isoform ratios [31] and in colorectal cancer [32].

*VEGFA* encodes VEGF-A, a key driver of angiogenesis, vascular permeability, and endothelial survival. VEGF-A signals predominantly through VEGFR2 (KDR/Flk-1), whereas VEGFR1 (FLT1) is commonly described as modulating ligand availability and overall signalling output [33]. In the kidney, VEGF-A signalling contributes to endothelial homeostasis and regulation of the glomerular filtration barrier [13]. The human *VEGFA* gene is located on chromosome 6p21.1 and comprises nine exons [34]. Regulatory SNPs in the *VEGFA* promoter and untranslated regions, including rs699947 (−2578C>A), rs833061 (−460T>C), and rs2010963 (5′ UTR), have therefore been extensively studied as candidate modifiers of diabetic microvascular complications [35,36]. The rs2010963 variant has shown genotype-dependent associations with circulating VEGF-A levels and susceptibility to DN in T2DM in a Chinese case–control study [37], while meta-analyses for diabetic retinopathy report mixed results [38,39].

The aim of this study was to evaluate the association of *ANGPT2* rs2442598 and *VEGFA* rs2010963 polymorphisms with DN in Slovenian patients with T2DM.

## 2. Materials and Methods

### 2.1. Patients

In this cross-sectional case–control study, we included 897 unrelated Caucasian subjects with T2DM from a Slovenian cohort. Participants were recruited at the outpatient clinics of the University Medical Centre Maribor and the General Hospitals of Murska Sobota and Slovenj Gradec. Based on nephrological evaluation, subjects were stratified into two groups: 344 patients with DN or diabetic kidney disease diagnosed according to the 1999 World Health Organization criteria, and 553 long-standing T2DM patients (≥10 years) without diabetic kidney disease (normoalbuminuria for 6 months) who served as controls. Normoalbuminuria was defined when the UACR was <3.0 mg/mmol in both sexes at the enrollment and again after 6 months (to determine chronicity of the status). Albuminuria was defined when the UACR was >3.0 mg/mmol in both sexes at the enrollment and again after 6 months.

Chronicity of albuminuria was confirmed by repeated urine testing. During enrolment, participants attended three laboratory visits within a 1–2-week period with collection of second morning urine samples. The same protocol was repeated after approximately six months. Persistent albuminuria was defined when albuminuria was present at enrolment and again after six months.

The diagnosis of T2DM was established in accordance with the guidelines of the American Diabetes Association.

After informed consent was obtained from all participants, venous blood samples were collected for biochemical analyses. The laboratory evaluation included hemoglobin A1c (HbA1c), total cholesterol, high-density lipoprotein (HDL), low-density lipoprotein (LDL), and triglycerides. Data on age, sex, blood pressure, duration of T2DM, hypertension and its duration, body mass index (BMI), smoking status, and the presence of T2DM-related microvascular complications, specifically diabetic retinopathy (DR) with its duration, DN, and estimated glomerular filtration rate (eGFR calculated on MDRD formula), were obtained using a standardized questionnaire. To minimise confounding by non-diabetic kidney disease, patients with overt renal disease unrelated to diabetes were excluded. Patients with poor glycaemic control, significant heart failure (New York Heart Association class II–IV), alcoholism, infection, and other causes of renal disease were also excluded.

The study protocol was approved by the Slovenian Medical Ethics Committee (No. 0120-105/2025).

### 2.2. Biochemical Analyses

Blood samples were processed using standard biochemical assays. For each participant, the albumin-to-creatinine ratio was measured in three urine specimens according to diagnostic guidelines. eGFR was calculated using the MDRD study equation based on serum creatinine; cystatin C was measured as an additional marker of kidney function.

### 2.3. Genotyping

Genomic DNA was isolated from 100 µL of peripheral blood using the QIAamp DNA Blood Mini Kit (Qiagen GmbH, Hilden, Germany). Genotyping of *ANGPT2* rs2442598 and *VEGFA* rs2010963 was performed using the StepOne™ 48-well real-time polymerase chain reaction (PCR) system (Applied Biosystems, Life Technologies, Foster City, CA, USA) together with TaqMan SNP Genotyping Assays (Applied Biosystems, Foster City, CA, USA), following the manufacturers’ protocols. Each 5 µL reaction contained 2.5 µL 2× Master Mix, 0.12 µL 40× Assay Mix, 1.88 µL DNase/RNase-free distilled water (Gibco, Invitrogen Life Technologies, Waltham, MA, USA), 0.5 µL of extracted genomic DNA, and VIC/FAM-labelled allele-specific probes/primers.

### 2.4. Statistical Analysis

All statistical analyses were performed using IBM SPSS Statistics for Windows, Version 29 (IBM Corp., Armonk, NY, USA). Hardy–Weinberg equilibrium was evaluated using a chi-square (χ^2^) goodness-of-fit test. Categorical variables, including genotype distributions, were compared between cases and controls using a chi-square test of independence. The Kolmogorov–Smirnov test was used to assess normality. Continuous variables were analysed with an unpaired Student’s *t*-test when normally distributed, or with a Mann–Whitney U-test for non-normal distributions.

Results are presented as mean ± standard deviation or as median with first and 3rd quartiles, as appropriate. Logistic regression was performed to evaluate the association of *ANGPT2* rs2442598 and *VEGFA* rs2010963 with DN in a case–control cohort with adjustment for waist circumference, systolic blood pressure (SBP), fasting glucose and triglycerides. Genetic association was analysed using co-dominant, additive, dominant, and recessive genetic models. Associations were expressed as odds ratios (ORs) with 95% confidence intervals (CIs). A *p*-value < 0.05 was considered statistically significant. To account for testing of two candidate SNPs (*ANGPT2* rs2442598 and *VEGFA* rs2010963), Bonferroni correction was applied, resulting in a corrected significance threshold of *p* < 0.025.

## 3. Results

Clinical and laboratory characteristics of the 897 participants with T2DM (344 DN cases; 553 controls without DN) are shown in Table 1. Compared with controls, DN cases had longer diabetes and hypertension duration, higher SBP, poorer glycaemic control, higher albuminuria, and lower eGFR (Table 1).

Genotype and allele distributions for *ANGPT2* rs2442598 are shown in Table 2. Associations were evaluated using a co-dominant genetic model (individual genotype comparisons) and allelic comparisons between cases and controls. In addition, additive, dominant, and recessive genetic models were analysed for this variant, defined with respect to the C allele. In the co-dominant model, individuals with the rs2442598 homozygous CC genotype had a significantly increased risk of DN compared with TT carriers (OR 1.86, 95% CI 1.06–3.26, *p* = 0.0303), whereas the TC genotype was not significantly associated with DN (OR 1.32, 95% CI 0.74–2.33, *p* = 0.34). In the additive genetic model, each additional C allele was associated with an increased risk of DN (OR 1.39, 95% CI 1.10–1.74, *p* = 0.0049), indicating a dose-dependent effect. Under the dominant model (CC vs. TT + TC), carriers of the CC genotype had a significantly higher risk of DN (OR 1.47, 95% CI 1.11–1.96, *p* = 0.0075). In contrast, the recessive model (TC + CC vs. TT) did not show a statistically significant association with DN (OR 1.59, 95% CI 0.92–2.75, *p* = 0.0935). At the allelic level, the C allele was more frequent among DN cases than in controls (74.6% vs. 69.0%, *p* = 0.0114). Genotype distributions for rs2442598 were consistent with the Hardy–Weinberg equilibrium in both cases and controls.

We also examined *VEGFA* rs2010963, and no significant association with diabetic nephropathy was observed in any of the tested genetic models (Table 3). Using the CC genotype as the reference, CG carriers had an adjusted OR of 1.14 (95% CI 0.69–1.88; *p* = 0.61), and GG carriers had an adjusted OR of 1.31 (95% CI 0.79–2.19; *p* = 0.30). In the additive model, the per-G-allele effect was not significant (OR 1.15, 95% CI 0.92–1.44; *p* = 0.22). Similarly, neither the dominant model (GG vs. CC + CG: OR 1.18, 95% CI 0.88–1.57; *p* = 0.26) nor the recessive model (CC vs. CG + GG: OR 1.21, 95% CI 0.75–1.97; *p* = 0.43) showed significant associations. At the allelic level, no significant difference in allele frequencies between cases and controls was observed (*p* = 0.824). Genotype distributions were consistent with the Hardy–Weinberg equilibrium in both cases and controls.

## 4. Discussion

This study evaluated *ANGPT2* rs2442598 and *VEGFA* rs2010963 in relation to DN in Slovenian patients with T2DM. We found a significant association for *ANGPT2* rs2442598, whereas *VEGFA* rs2010963 showed no evidence of association. Importantly, the association for *ANGPT2* rs2442598 remained significant in both the additive and dominant genetic models, supporting the robustness of this finding. To our knowledge, this is among the first studies to report an association between *ANGPT2* rs2442598 and diabetic nephropathy in patients with type 2 diabetes mellitus. Clinically, compared with controls, DN cases had longer diabetes and hypertension duration, higher systolic blood pressure, poorer glycaemic control, higher albuminuria, higher creatinine and cystatin C, and lower eGFR. These differences reflect established risk factors and complications of DN and support the robustness of phenotypic classification in our cohort, providing an appropriate clinical context for the genetic analyses. The key genetic finding was a significant association between *ANGPT2* rs2442598 and DN, with increased risk in C-allele carriers and the strongest effect in CC homozygotes in the co-dominant model (CC vs. TT: OR 1.86, 95% CI 1.06–3.26; *p* = 0.030). In addition, the additive model demonstrated a significant per allele effect (OR 1.39, 95% CI 1.10–1.74; *p* = 0.0049), while the dominant model (CC vs. TT+TC) also showed a significant association (OR 1.47, 95% CI 1.11–1.96; *p* = 0.0075). Genotype distributions were consistent with the Hardy–Weinberg equilibrium in both groups. Notably, the minor allele for rs2442598 in our population was T, indicating that DN risk is associated with the common (major) C allele at this locus. This suggests that DN susceptibility may be influenced by common regulatory genetic variation rather than rare alleles with large effects. The consistency of association across multiple genetic models further supports the robustness of the observed genetic effect.

In contrast, *VEGFA* rs2010963 showed no evidence of association with DN across genotype and allele comparisons. Consistently, no association was observed in the additive (per-G-allele OR 1.15, 95% CI 0.92–1.44; *p* = 0.22), dominant (OR 1.18, 95% CI 0.88–1.57; *p* = 0.26), or recessive (OR 1.21, 95% CI 0.75–1.97; *p* = 0.43) genetic models. Together, these data suggest that rs2442598 at the *ANGPT2* locus may contribute to DN susceptibility in Slovenian patients with T2DM, whereas rs2010963 in *VEGFA* does not show a detectable effect in this cohort.

Genetic association evidence for *ANGPT2* in DN remains limited, and rs2442598 has not been directly investigated in DN-specific cohorts to date. Badr et al. reported that rs2442598 was associated with higher odds of ESKD, although ESKD was analysed irrespective of etiology [40]. In Brazilian patients with type 1 diabetes, Dieter et al. found that the rs2442598 A allele and A/A genotype were more frequent in DR, with the A/A genotype remaining associated with DR after multivariable adjustment [28]. In a Han Chinese case–control study, Lan et al. reported that the rs2442598 TT genotype was associated with lower coronary artery disease risk [29]. Outside diabetes-specific DN studies, *ANGPT2* variants, including rs2442598, have also been examined in other vascular and inflammatory conditions, supporting a broader link to microvascular dysfunction [30,31,32]. For other *ANGPT2* variants, evidence in DN is limited; however, He et al. reported that the *ANGPT2* 1233A/G polymorphism was associated with T2DM and DN, and that the G allele was linked to DN risk when patients were grouped by urinary albumin excretion rate (UAER) [41].

Independent of genetic association studies, multiple clinical and translational reports show that circulating and/or urinary Ang-2 is elevated in DKD and correlates with albuminuria and markers of disease severity, consistent with Ang-2 and Tie2 pathway dysregulation in glomerular microvascular injury [25,42,43,44]. These observations provide biological plausibility for our genetic findings and support a contributory role of *ANGPT2* dysregulation in DN pathogenesis.

Evidence for *VEGFA* rs2010963 varies across studies. In a Han Chinese case–control study, Luo et al. reported higher DN risk in carriers of the C allele (GC/CC) compared with GG homozygotes [37]. In an Iranian T2DM cohort, *VEGFA* rs2010963 showed a higher GG genotype frequency in patients with microalbuminuria, while the G allele alone was not associated with microalbuminuria [45]. In a Sudanese T2DM case–control study, rs2010963 was associated with DN and DR, and the GC genotype and G allele were associated with DR risk [46]. Vailati et al. reported higher retinal *VEGFA* gene expression (mRNA) in rs2010963 C-allele carriers (GC/CC) compared with GG homozygotes [47]. However, an updated meta-analysis by Lu et al. found no significant overall association between rs2010963 and DR across genetic models [39].

In macrovascular disease, rs2010963 has been linked to myocardial infarction in T2DM in a Slovenian cohort, with higher risk in C-allele carriers (CC/CG) than in GG [48]. A coronary heart disease meta-analysis also reported an rs2010963 association with myocardial infarction risk [49]. Other *VEGFA* polymorphisms linked to DN include rs833061, which has been associated with DN risk in T2DM in a meta-analysis [50], and the *VEGFA* locus rs6921438, which has been associated with DN in Caucasian T2DM [51].

Mechanistically, Ang-1 activates Tie2 to stabilize endothelial junctions, reducing plasma leakage and limiting VEGF-A–induced hyperpermeability [20,21]. Ang-2 reduces Ang-1–Tie2 barrier signaling and activates β1-integrins, promoting endothelial destabilisation and vascular leak [20,22,24]. In DKD, glomerular Ang–Tie2 pathway activation has been linked to disease progression [19]. Podocytes are a major source of glomerular VEGF-A that signals to glomerular endothelial cells. VEGF-A supports endothelial survival, yet dysregulated podocyte VEGF-A under hyperglycaemia can induce proteinuria and glomerular microvascular injury [52,53]. Similarly, increased Ang-2 expression in podocytes can directly disrupt the glomerular filtration barrier, inducing proteinuria and promoting glomerular endothelial apoptosis [54]. Taken together, our genetic findings, in conjunction with existing experimental and clinical evidence, support a model in which increased ANGPT2 activity contributes to glomerular endothelial destabilisation and albumin leakage in DN. An important limitation of our study is that, despite a moderate overall sample size (cases *n* = 344; controls *n* = 553), some genotype categories were infrequent, which may reduce power to detect small effect sizes, recessive effects, or gene–gene interactions and may widen confidence intervals. Our analysis was restricted to two candidate polymorphisms, and additional variants within *ANGPT2*, *VEGFA*, or related angiogenic pathways may also contribute to DN susceptibility. The study population was limited to Slovenian patients with T2DM, so replication in independent cohorts and other populations is needed to support external validity. As a case–control study, residual confounding and phenotype misclassification remain possible, and results should be interpreted with multiple comparisons in mind. Future studies integrating genotype with circulating or urinary Ang-2 and VEGF-A levels and, where available, renal tissue expression data would help clarify underlying mechanisms.

## 5. Conclusions

In conclusion, this study demonstrates a significant association between the *ANGPT2* rs2442598 polymorphism and DN in Slovenian patients with type 2 diabetes mellitus, whereas no association was observed for *VEGFA* rs2010963. These findings support a contributory role of *ANGPT2* genetic variation in DN susceptibility and align with accumulating experimental and clinical evidence implicating Ang-2–Tie2 pathway dysregulation in diabetic kidney disease. Although replication in independent cohorts and functional studies is required, our results highlight *ANGPT2* as a biologically plausible genetic factor in DN pathophysiology.

## Figures and Tables

**Table 1:** Clinical characteristics and laboratory characteristics of T2DM (type 2 diabetes mellitus), patients with diabetic nephropathy (DN; cases) and without DN (controls).

	Cases (N = 344)	Controls (N = 553)	*p* Value
Sex (M)	201 (58.4%)	318 (57.5%)	0.78
Age (years)	65.3 ± 9.4	65.4 ± 8.6	0.90
Duration of T2D (years)	16.0 (13.0–20.0)	15.0 (12.0–19.0)	***<0.001***
Duration of hypertension (years)	12.0 (7.0–18.0)	11.0 (5.0–16.0)	***0.0058***
SBP (mmHg)	155.0 (142.7–167.0)	145.0 (135.0–160.0)	***<0.001***
DBP (mmHg)	85.0 (77.0–90.0)	80.0 (75.0–90.0)	*0.0759*
BMI	30.39 ± 3.56	29.94 ± 3.91	*0.08*
Waist circumference	108.56 ± 10.41	104.59 ± 11.78	***<0.001***
Active smokers	32 (9.3%)	70 (12.7%)	*0.12*
CVD	89 (25.9%)	165 (29.8%)	*0.20*
DR	149 (43.3%)	115 (20.8%)	***<0.001***
Duration of DR (years)	5.0 (5.0–6.0)	6.0 (4.0–8.0)	***<0.001***
Dneuropathy	69 (20.1%)	39 (7.1%)	***<0.001***
S-HbA1c (%) ^1^	7.70 (6.90–8.72)	7.50 (6.90–8.20)	***<0.001***
S-fasting glucose (mmol/L)	8.70 (7.18–10.33)	8.10 (6.80–9.50)	***<0.001***
S-Hb (g/L)	138.87 ± 13.26	139.65 ± 11.84	*0.36*
S-urea (mmol/L)	6.20 (5.20–7.70)	5.90 (4.80–7.40)	***0.0049***
S-creatinine (μmol/L)	80.0 (68.0–102.0)	77.0 (65.0–90.0)	***0.0025***
Male sex	89.0 (73.0–106.0)	84.0 (71.0–98.0)	***0.0025***
Female sex	72.0 (58.0–88.0)	70.0 (61.0–80.0)	*0.097*
eGFR (MDRD equation, mL/min)	60.0 (58.0–64.0)	77.0 (60.0–88.0)	***<0.001***
Male sex	60.0 (60.0–60.0)	79.0 (68.0–87.0)	***<0.001***
Female sex	62.0 (59.0–66.0)	60.0 (60.0–60.0)	***<0.001***
S-cystatin C (mg/L)	0.82 (0.69–1.03)	0.74 (0.65–0.86)	***<0.001***
S-Total cholesterol (mmol/L)	4.35 (3.80–5.20)	4.40 (3.90–5.10)	*0.82*
S-HDL (mmol/L)	1.20 (1.00–1.40)	1.20 (1.00–1.40)	*0.86*
S-LDL (mmol/L)	2.40 (2.00–3.00)	2.40 (2.00–3.00)	*0.66*
S-TGS (mmol/L)	1.60 (1.10–2.32)	1.40 (1.00–2.10)	***0.0123***
U-albumin/creatinine ratio (g/mol), sampleno.1	7.92 (3.51–22.67)	1.00 (0.60–1.58)	***<0.001***
U-albumin/creatinine ratio (g/mol), sampleno.2	7.97 (3.58–24.51)	1.03 (0.68–1.70)	***<0.001***
U-albumin/creatinine ratio (g/mol), sampleno.3	8.18 (3.52–22.82)	1.02 (0.68–1.70)	***<0.001***

Statistically significant values are written in bold. Legend: DN: diabetic nephropathy; T2DM: Type 2 diabetes mellitus; SBP: Systolic blood pressure; DBP: Diastolic blood pressure; BMI: Body mass index; CVD: Cardiovascular disease; DR: Diabetic retinopathy; S-HbA1c (%) ^1^: Glycated hemoglobin; Hb: serum hemoglobin; eGFR: estimated glomerular filtration rate; S-TGS: triglyceride; HDL: High-density lipoprotein; LDL: Low-density lipoprotein.

**Table 2:** Distribution of *ANGPT2*_rs2442598 genotypes and alleles in the case group (patients with T2DM (type 2 diabetes mellitus) and DN (diabetic nephropathy)) and control group (patients with T2DM without DN). Logistic regression analysis was used.

*ANGPT2* rs2442598	Case (N = 344)	Control (N = 553)	*p* Value	adj OR (95% CI)	*p* Value for OR
TT	21 (6.1%)	52 (9.4%)	***0.0389***	*ref*	
TC	133 (38.7%)	239 (43.2%)		*1.32 (0.74–2.33)*	*p = 0.34*
CC	190 (55.2%)	262 (47.4%)		***1.86 (1.06–3.26)***	***p = 0.0303***
ADDITIVE					
per C allele				***1.39 (1.10–1.74)***	***p = 0.0049***
DOMINANT					
TT + TC	154 (44.8%)	291 (52.6%)	***0.0222***	*ref.*	
CC	190 (55.2%)	262 (47.4%)		***1.47 (1.11–1.96)***	***p = 0.0075***
RECESSIVE					
TT	21 (6.1%)	52 (9.4%)	*0.0789*	*ref.*	
TC + CC	323 (93.9%)	501 (90.6%)		*1.59 (0.92–2.75)*	*p = 0.0935*
ALLELES					
T (MAF)	175 (25.4%)	343 (31.0%)	***0.0113***	*ref*	
C	513 (74.6%)	763 (69.0%)		1.32 (1.06–1.63)	***p* = 0.0114**
HWE (*p* value)	0.72	0.81			

Statistically significant values are written in bold. Adjusted ORs from logistic regression controlling for waist circumference, systolic blood pressure, fasting glucose, and triglycerides. Genetic models test for effects of the C (risk) allele. Associations in the additive (*p* = 0.0049) and dominant (*p* = 0.0075) models remain significant after Bonferroni correction for 2 SNPs (α = 0.025). Legend: MAF—minor allele frequency; HWE—Hardy–Weinberg equilibrium; OR—odds ratio; CI—confidence interval.

**Table 3:** Distribution of *VEGFA* rs2010963 genotypes and alleles in the case group (patients with T2DM (type 2 diabetes mellitus) and DN (diabetic nephropathy)) and control group (patients with T2DM without DN). Logistic regression analysis was used.

*VEGFA* rs2010963	Case (N = 344)	Control (N = 553)	*p* Value	adj OR (95% CI)	*p* Value for OR
CC	32 (9.3%)	55 (9.9%)	*0.951*	*ref*	
CG	167 (48.5%)	267 (48.3%)		*1.14 (0.69–1.88)*	*p = 0.61*
GG	145 (42.2%)	231 (41.8%)		*1.31 (0.79–2.19)*	*p = 0.30*
ADDITIVE					
per G allele				*1.15 (0.92–1.44)*	*p = 0.22*
DOMINANT					
CC + CG	199 (57.8%)	322 (58.2%)	*0.91*	*ref.*	
GG	145 (42.2%)	231 (41.8%)		*1.18 (0.88–1.57)*	*p = 0.26*
RECESSIVE					
CC	32 (9.3%)	55 (9.9%)	*0.75*	*ref.*	
CG + GG	312 (90.7%)	498 (90.1%)		*1.21 (0.75–1.97)*	*p = 0.43*
ALLELES					
C (MAF)	231 (33.6%)	377 (34.1%)	*0.824*	*ref.*	
G	457 (66.4%)	729 (65.9%)		1.02 (0.84–1.25)	*p* = 0.82
HWE (*p* value)	0.40	0.42			

Adjusted ORs from logistic regression controlling for waist circumference, systolic blood pressure, fasting glucose, and triglycerides. Genetic models test for effects of the G (risk) allele. Associations in all models remain unsignificant after Bonferroni correction for 2 SNPs (α = 0.025). Legend: MAF—minor allele frequency; HWE—Hardy–Weinberg equilibrium; OR—odds ratio; CI—confidence interval.

## Data Availability

The data presented in this study are available on request from the corresponding author due to sensitive information (patients’ clinical data).
